# Fuscan: a robust DNA fusion caller for targeted sequencing data in cancer diagnostics

**DOI:** 10.1093/bioadv/vbag152

**Published:** 2026-05-29

**Authors:** Zhaoying Liu, Siyu Wang, Si Chen, Haiyan Feng, Xiao Hu, Ping Zhou, Dong Shi

**Affiliations:** Shanghai Yijian Medical Laboratory Co., Ltd., Shanghai, 201802, China; Shanghai Yijian Medical Laboratory Co., Ltd., Shanghai, 201802, China; Shanghai Yijian Medical Laboratory Co., Ltd., Shanghai, 201802, China; Department of Pathology, The First Hospital of Zibo, Zibo, Shandong, 255200, China; Department of Pathology, The First Hospital of Zibo, Zibo, Shandong, 255200, China; Department of Pathology, The First Hospital of Zibo, Zibo, Shandong, 255200, China; Shanghai Yijian Medical Laboratory Co., Ltd., Shanghai, 201802, China

## Abstract

**Motivation:**

Gene fusions resulting from genomic structural variation in somatic cells have been increasingly identified as central events driving oncogenesis. Ultra-deep targeted sequencing of driver fusions informs therapeutic selection in precision oncology. However, most structural variant (SV) callers were primarily architected for whole genome sequencing, failing to resolve the artifacts and alignment errors that drive false-positive calls in high-depth targeted data.

**Results:**

Here, we describe Fuscan, a robust DNA fusion caller specifically optimized for targeted sequencing data to identify oncogenic drivers. Fuscan improves sensitivity by focusing alignment on targeted driver sequences while simultaneously filtering homologous genomic regions to prevent false-positive partner-gene breakpoints. We performed targeted sequencing on 85 non-small cell lung cancer clinical specimens (comprising tissue and body fluids), four SV reference standards at 0.5% allele frequency, and 282 healthy-control leukocyte samples. We benchmarked Fuscan against established SV callers, achieving an area under the curve (AUC) of 0.992 and demonstrating its robustness in challenging clinical scenarios, including low-tumor-content tissues and liquid biopsies.

**Availability and implementation:**

Fuscan is available on our GitHub repository: https://github.com/YJmedLab/Fuscan.

## 1 Introduction

Gene fusions have been increasingly confirmed as the driving force of oncogenesis in several cancer types. Many recurrent fusions have been identified as molecular targets for therapy, such as *ALK*, *ROS1*, or *RET* in non-small cell lung cancer (NSCLC) (Soda *et al.* 2007, Kohno *et al.* 2012, Takeuchi *et al.* 2012, Qiu *et al.* 2025). The expanding repertoire of U.S. Food and Drug Administration (FDA)-approved targeted therapies for NSCLC enables the treatment of these oncogenic fusions, as exemplified by crizotinib, alectinib, and lorlatinib for *ALK* rearrangements (Solomon *et al.* 2014), entrectinib for *ROS1*, and selpercatinib and pralsetinib for *RET* (Rodak *et al.* 2021). To address low tumor cellularity in tissue and scant circulating tumor DNA (ctDNA) in liquid biopsies, high-depth probe-capture sequencing is utilized to ensure robust companion diagnostics and the identification of actionable therapeutic targets (Bruno and Fontanini, 2020). However, specialized algorithms optimized for DNA fusion detection in targeted sequencing data remain limited, such as FACTERA and Genefuse. FACTERA identifies fusions by leveraging discordant and soft-clipped reads within standard alignment outputs (Newman *et al.* 2014). Conversely, Genefuse bypasses whole-genome alignment, instead prioritizing the direct identification of reads that span pre-defined gene pairs (Pan *et al.* 2024). While originally engineered for whole genome sequencing (WGS), DELLY has been successfully adapted for targeted sequencing, notably as the structural variant (SV) caller pipeline for MSK-IMPACT, which is the first FDA-authorized next-generation sequencing (NGS) panel for solid tumors (Cheng *et al.* 2015). The algorithm delineates diverse rearrangement classes by integrating discordant paired-end orientations with split-read alignments (Rausch *et al.* 2012). Manta utilizes a graph-based assembly framework for rapid SV and indel discovery (Chen *et al.* 2016). GRIDSS2 provides high-resolution characterization of rearrangements through single-breakend phasing (Cameron *et al.* 2021). While initially optimized for WGS, both tools support targeted sequencing data.

However, these approaches exhibit deficiencies in sensitivity, specificity, and handling of false fusion signals in clinical detection. Pan *et al.* (2024) modified FACTERA to input the targeted fusion regions one at a time rather than all at once and added 177 additional fusion genes and partners sourced from published studies and the COSMIC database to Genefuse’s original file to improve the sensitivity of both tools. Despite conferring 80.8% and 76.9% sensitivities on FACTERA and Genefuse, respectively, the alterations above failed to detect certain fusion events. Genefuse is restricted to predefined gene sets, which may fail to capture the high variability of fusion partners and breakpoints (Jiang *et al.* 2018). Previous studies indicated that DELLY exhibits high sensitivity (100%) but low specificity (0.98%), resulting in numerous false-positive candidate fusions (Newman *et al.* 2014). In clinical fusion detection for oncology, insufficient sensitivity can lead to patients missing therapeutic opportunities, while low specificity can result in false positives, interfere with treatment strategies (Lindeman *et al.* 2018, Merker *et al.* 2018). Low tumor cellularity and scant ctDNA provide sparse fusion signals that also challenge the sensitivity and specificity of detection within high-depth sequencing data.

Here, we developed a novel fusion detection tool for DNA sequencing data named Fuscan. Fuscan is optimized for high-depth targeted sequencing by restricting alignment to probe-covered regions. It utilizes a baseline derived from 20 normal controls to filter false positives, eliminating the requirement for matched normal samples. Benchmarking against clinical data and reference standards confirms its robustness, particularly for low-cellularity tissues and scant ctDNA in plasma.

## 2 Methods

### 2.1 Fuscan overview

**Figure 1:**
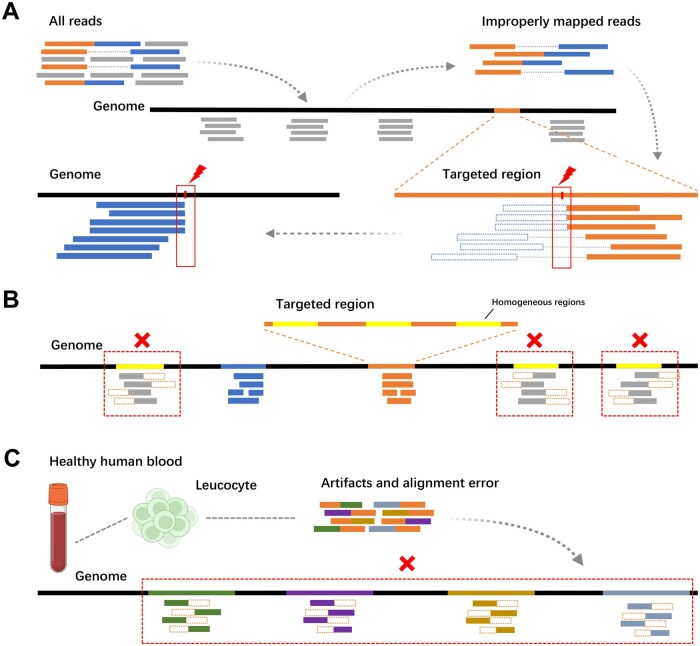
Overview of Fuscan analysis and filtering workflow. (A) Workflow of Fuscan breakpoints analysis, including main steps of whole-genome alignment, local alignment, and breakpoints identification. (B) A filtering scheme based on homologous genes. The boxed region refers to the part of the genome that is homologous to the targeted region, and breakpoints located at this position are filtered out. (C) A filtering approach based on a background established by healthy-individual controls.

As shown in Fig. 1A, Fuscan’s workflow consists of the following steps: (i) Take all reads for alignment to the reference genome and collect improperly mapped reads (discordant reads and split reads). (ii) Align all improperly mapped reads to the sequence of targeted regions and search for potential fusion reads (discordant paired-end reads and soft-clipped reads). (iii) Extract sequences from putative partners within potential fusion reads and align them to the reference genome. (iv) Exclude the positions of homologous regions and the background baseline to identify putative breakpoint loci. All homologous regions are obtained from the BLAST-like alignment tool (BLAT) alignment of the targeted region sequences against the genome sequence (Fig. 1B). The background baseline for noise filtering is established by identifying commonly occurring background breakpoints through the same analysis protocol on leukocytes from healthy individuals (Fig. 1C).

### 2.2 Data preparation and processing

To compare the performance of Fuscan with other approaches, we applied targeted sequencing of 85 samples comprising tissue specimens and body fluid from NSCLC patients, 4 SV formalin-fixed paraffin-embedded (FFPE) reference standards with 0.5% mutation frequency, and 282 leukocyte samples from healthy individuals. All patient samples were polymerase chain reaction (PCR)-verified as positive for *ALK*, *ROS1*, or *RET* fusions. The cohort consisted of 64 FFPE tumor tissues and 21 liquid biopsy samples (13 plasma and 8 pleural effusion). The 0.5% SV FFPE reference standards were prepared by mixing 90% of the NA12878 reference human genome (GW-SHF001, GeneWell Biotechnology Co., Ltd) with 10% of 5% SV FFPE reference standards (GW-OPSM002, GeneWell Biotechnology Co., Ltd; CBP90010, Nanjing Cobioer Gene Technology Co., Ltd.). To evaluate the pan-cancer performance of Fuscan, 20 additional fusion-positive samples (18 FFPE and 2 plasma) were included across multiple malignancies (CRC, BLCA, PRAD, STAD, CESC, KIRC, and LIHC) harboring fusions such as *FGFR2*, *TMPRSS2*, and *NTRK1*. The panel was designed to cover multiple hot-spot regions in cancer, including genes such as *EGFR*, *BRAF*, and *KRAS*, as well as recurrent fusion genes (e.g. *ALK*, *ROS1*, *RET*, and *NTRK1*). Probe region design for fusion detection covering full-length gene exons and certain introns. All sequencings were performed using YJSeq300Dx with PE150. The median average sequencing depth was 695× for tissue and WBC samples, 2059× for the reference standard, and 11 337× for liquid biopsy samples. Detailed sample information and data QC summaries are available in Table 1, available as supplementary data at *Bioinformatics Advances* online.

**Table 1:** The results of 12 fusion events in the reference standards using Fuscan, DELLY, FACTERA, Genefuse, GRIDSS2, and Manta.

Sample	Fusion	Fuscan	Genefuse	FACTERA	DELLY	GRIDSS2	Manta
**RS1_1**	*EML4*(13)-*ALK*(20)	Detected	Not detected	Detected	Detected	Not detected	Detected (FP)
**RS1_1**	*CCDC6*(1)-*RET*(12)	Detected	Detected	Detected	Detected	Not detected	Detected (FP)
**RS1_2**	*EML4*(13)-*ALK*(20)	Detected	Not detected (FP)	Detected (FP)	Detected (FP)	Detected (FP)	Not detected
**RS1_2**	*CCDC6*(1)-*RET*(12)	Detected	Detected (FP)	Detected (FP)	Detected (FP)	Detected (FP)	Not detected
**RS1_3**	*EML4*(13)-*ALK*(20)	Detected	Not detected	Detected	Detected	Detected (FP)	Not detected
**RS1_3**	*CCDC6*(1)-*RET*(12)	Detected	Detected	Not Detected	Detected	Detected (FP)	Not detected
**RS2_1**	*EML4*(6)-*ALK*(20)	Detected	Detected	Detected	Detected	Detected (FP)	Not detected
**RS2_1**	*CD74*(6)-*ROS1*(34)	Detected	Detected	Detected	Detected	Detected (FP)	Not detected
**RS2_2**	*EML4*(6)-*ALK*(20)	Detected	Not detected	Detected	Detected	Detected (FP)	Not detected
**RS2_2**	*CD74*(6)-*ROS1*(34)	Detected	Detected	Detected	Detected	Detected (FP)	Not detected
**RS2_3**	*EML4*(6)-*ALK*(20)	Detected	Detected	Detected	Detected	Detected (FP)	Not detected
**RS2_3**	*CD74*(6)-*ROS1*(34)	Detected	Detected	Detected	Detected	Detected (FP)	Not detected

FP, false-positive fusion events.

### 2.3 Filter profiles preparing

Fuscan requires paired-end FASTQ files and a BED file specifying regions of interest for fusion genes. If the BED file is reused, users can employ the Fuscan prepare script to generate a PKL file containing information on homologous genes. The PKL file can replace the BED file as input, negating the need for repeated homologous gene identification. Homologous gene analysis is performed with BLAT (v.39x1) (Kent 2002) using the parameters “-stepSize 5 -minIdentity 90 -minScore 20.” Fuscan also offers a background construction feature that enables users to conduct fusion detection on samples from healthy individuals. The building script generates a PKL file containing the positions of background loci.

### 2.4 Improperly mapped reads alignment

To collect improperly mapped reads, Fuscan first aligns all reads to the reference genome sequences using BWA-mem (0.7.18) (Li and Durbin 2009) with the parameter “-L [60,60].” Due to the high penalty for clipping, Fuscan extracts reads with a mapping quality (MAPQ) of less than 30 as improperly mapped and reads with an insert size greater than 1000 bp or mapped to different chromosomes. All improperly mapped reads are aligned to sequences of targeted regions provided from the BED file using BWA-mem with parameters “-B 13 -O [18,18].” The alignment and subsequent analysis of targeted regions are processed in parallel to optimize analysis speed. The soft-clipped part sequences and unmapped reads whose mate was mapped well in the targeted region (discordant reads) are extracted for whole-genome alignment to identify the genomic locations of potential partner genes.

### 2.5 Breakpoints merging and filtering

The targeted region and the partner genes in the genome are considered to have potential breakpoints at positions between the soft-clipped and mapped parts. Discordant reads must support potential breakpoints within 100 bp. Breakpoints within 10 bp are merged, and the number of supporting reads (split reads and discordant reads) is counted. Fuscan filters potential breakpoints based on the aforementioned filter profiles for homologous genes and the background. All breakpoints are annotated using SnpEff (4.3t) (Cingolani *et al.* 2012). Users can provide specific fusion gene pairs to Fuscan and set their specific thresholds for the number of supporting split and discordant reads. Additionally, breakpoints located in introns, exons, and intergenic regions can have separate filtering thresholds. Given that most clinically actionable fusions occur in intronic regions(Rosenbaum *et al.* 2018, Cui *et al.* 2020, Wu *et al.* 2023), Fuscan employs region-specific thresholds for breakpoint detection. While the thresholds for split reads and discordant reads across different genomic regions (introns, exons, and intergenic regions) are fully customizable.

### 2.6 Assessment of comprehensive performance

We compared the comprehensive performance of Fuscan with other fusion detection tools, including FACTERA (1.4.4), DELLY (v1.3.3), Genefuse (v0.8.0), GRIDSS2 (2.13.2), and Manta (1.6.0). The evaluation focused on sensitivity in low-frequency reference standards, sensitivity and specificity in clinical samples, and the running time of parallel optimization. For clinical samples, we manually confirmed false-positive fusions using IGV. We designed a BED file covering the genomic regions targeted by probes for *ALK*, *ROS1*, and *RET* fusions, as input for targeted regions in Fuscan, such as intron 19 of *ALK*, intron 33 of *ROS1*, and intron 11 of *RET* (Table 1, available as supplementary data at *Bioinformatics Advances* online). Notably, this BED file also served as input for FACTERA and was used to filter the results from DELLY, Genefuse, GRIDSS2, and Manta. Fuscan and Genefuse require paired FASTQ files as input, while FACTERA, DELLY, GRIDSS2, and Manta require deduplicated BAM files. Reads were aligned to the hg19 using BWA-MEM (v0.7.18) with default parameters. Subsequently, PCR duplicates were marked and removed using Picard tools (2.20.2). Genefuse used its own list of coordinated target genes as input. FACTERA, DELLY, and GRIDSS2 were run with default parameters, and we filtered DELLY results tagged as “LowQual.” In the absence of matched normal controls, Manta was operated in tumor-only mode, utilizing the--exome flag to bypass default high-depth filtering. For subsequent analysis, we constructed a background library for Fuscan using leukocyte samples from 20 healthy individuals. These samples also served as the Panel of Normals (PoN) for other tools; fusion candidates with concordant breakpoints were flagged during benchmarking. To validate Fuscan’s performance on an external dataset with a different panel, we retrieved data from 104 fusion-positive leukemia samples(Chen *et al.* 2025). Data processing was identical to that of our clinical cohort, with the sole exception of omitting the PoN. We selected 12 samples with data sizes ranging from 1.2 GB to 14.0 GB to evaluate the analysis time of all tools using 8 processes, and tested 10 GB of sequencing data and increased the number of processes from 8 to 80 to measure the computational time of these tools.

## 3 Results

### 3.1 Benchmarking Fuscan on reference standards

To assess the detection performance of Fuscan relative to DELLY, FACTERA, Genefuse, GRIDSS2, and Manta in ultra-low frequency DNA fusion samples, we utilized two different SV reference standards at 0.5%. One of the standards contains the fusions *EML4*(6)-*ALK*(20) and *CD74*(6)-*ROS1*(34), while the other contains *EML4*(13)-*ALK*(20) and *CCDC6*(1)-*RET*(12). The reference standards were subjected to triplicate technical replicates, and all 12 fusion events were confirmed by droplet digital PCR (ddPCR). The median average sequencing depth of these samples was 2059×. As shown in Table 1 (Table 2, available as supplementary data at *Bioinformatics Advances* online), Fuscan and DELLY detected all fusion events (12/12), followed by FACTERA (11/12). GRIDSS2 and Genefuse both identified eight events, while Manta detected only two events. In one of the technical replicates, DELLY, FACTERA, and Genefuse detected true-positive results with additional false-positive fusions. Notably, both GRIDSS2 and Manta yielded additional false-positive calls among the detected samples. Despite the application of PoN-based filtering, the impact on these tools was negligible (Table 2, available as supplementary data at *Bioinformatics Advances* online). The false-positive fusions requires manual intervention for review in clinical detection, affecting the automation process and increasing the risk of erroneous clinical diagnosis. GRIDSS2, FACTERA, Genefuse, and particularly Manta, failed to detect several fusion events. Due to the low levels of ctDNA in some clinical blood samples or the low tumor content in tissue samples, the detection capabilities of these tools may be limited in such application scenarios.

**Table 2:** The benchmarking results for Fuscan, DELLY, FACTERA, Genefuse, GRIDSS2, and Manta in clinical samples.

Methods	Sensitivity	Specificity	Area Under the Curve (AUC)
**Fuscan**	100% (85/85)	99.45% (363/365)	0.992
**Genefuse**	78.82% (67/85)	96.71% (353/365)	0.911
**FACTERA**	88.24% (75/85)	96.43% (352/365)	0.915
**DELLY**	100% (85/85)	65.20% (238/365)	0.974
**GRIDSS2**	96.47% (82/85)	18.35% (67/365)	0.916
**Manta**	85.9% (73/85)	11.23% (41/365)	0.932

### 3.2 Benchmarking Fuscan on clinical samples

**Figure 2:**
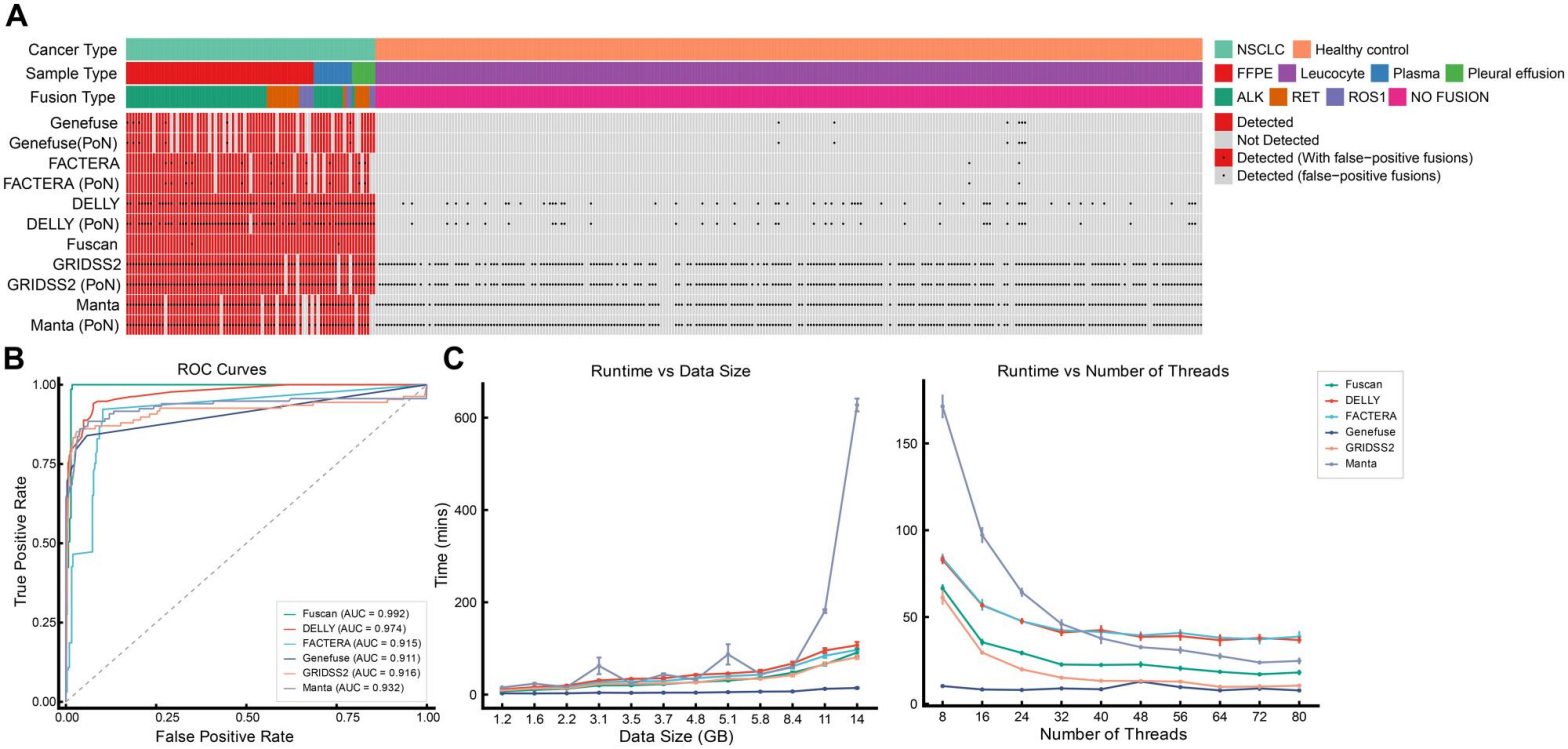
Fusion detection results of clinical samples and running time for each fusion detection tool. (A) Fusion detection performance across all detection tools. Top annotations indicate cancer type, sample type, and fusion type. Detection status is color-coded as follows: gray, not detected; red, detected; red with a dot, detected with false positives; gray with a dot, false positives only. (B) ROC curves for all evaluated tools. (C) Running time for each tool.

To evaluate the performance of Fuscan and alternative tools on clinical specimens, we assessed 85 samples from NSCLC patients (tissue and body fluids) and 282 leukocyte samples from healthy individuals (Fig. 2A, Table 2, Table 3, available as supplementary data at *Bioinformatics Advances* online). The median average sequencing depth was 695× for tissue and WBC samples, and 11 337× for liquid biopsy samples. Defining only calls passing internal quality control as detected, Fuscan and DELLY showed perfect concordance with reference standards, achieving 100% (85/85) sensitivity. In contrast, FACTERA and Genefuse reached sensitivities of 88.24% (75/85) and 78.82% (67/85), respectively. Among the events missed by Genefuse, four partner genes (*MTA3*, *KIF13A*, *IL1R2*, and *ALOX5*) had breakpoints outside its targeted gene list. GRIDSS2 and Manta achieved sensitivities of 96.47% (82/85) and 85.9% (73/85), respectively. Of the 85 clinical samples, 30 featured low tumor abundance (17 FFPE with <5% tumor content and 13 plasma samples); in this challenging subset, only Fuscan and DELLY successfully detected all events.

Using leukocyte samples as negative controls and counting any additional false positive in positive samples, Fuscan achieved a specificity of 99.45% (363/365), followed by Genefuse at 96.71% (353/365), FACTERA at 96.43% (352/365), DELLY at 65.20% (238/365), GRIDSS2 at 18.35% (67/365), and Manta at 11.23% (41/365). PoN filtering showed no impact on any tool except DELLY. DELLY’s specificity improved to 72.05% (263/365) after filtering, but one true positive was lost in the process.

To evaluate comprehensive performance, receiver operating characteristic (ROC) curves were generated using tool-specific metrics (e.g. improper ratio for Fuscan, RV+SV for DELLY, and QUAL for GRIDSS2) of all fusion events. True fusion events that were not detected by the software were assigned a value of zero. As shown in Fig. 2B, Fuscan achieved the highest AUC (0.992, 95% CI 0.984–1.000), followed by DELLY (0.974, 95% CI 0.960–0.987), GRIDSS2 (0.916, 95% CI 0.872–0.961), FACTERA (0.915, 95% CI 0.885–0.944), Genefuse (0.911, 95% CI 0.875–0.947), and Manta (0.932, 95% CI 0.906–0.959). We further evaluated the pan-cancer robustness of Fuscan using 20 additional fusion-positive samples (18 FFPE and 2 plasma) across seven malignancies (CRC, BLCA, PRAD, STAD, CESC, KIRC, and LIHC). These samples harbored diverse driver fusions, including *FGFR2*, *TMPRSS2*, and *NTRK1*, all of which were successfully identified by Fuscan (Table 4, available as supplementary data at *Bioinformatics Advances* online).

### 3.3 Benchmarking Fuscan on an independent leukemia cohort

To assess Fuscan’s generalizability across different panel designs, we analyzed a DNA sequencing dataset of 104 leukemia samples (bone marrow or peripheral blood) with gene fusions confirmed by qRT-PCR or NGS (Chen *et al.* 2025). We defined Fuscan’s targeted regions based on its panel (Table 1, available as supplementary data at *Bioinformatics Advances* online), which covers 95 introns across 26 genes. Benchmarking demonstrated that Fuscan achieved the highest sensitivity at 96.2% (100/104), outperforming GRIDSS2 (92.3%, 96/104), Manta (90.4%, 94/104), FACTERA (80.7%, 84/104), DELLY (77.8%, 80/104), and Genefuse (56.7%, 59/104) (Table 5, available as supplementary data at *Bioinformatics Advances* online). Notably, Fuscan successfully recovered two of the four fusions that were detected by PCR but missed by NGS in the original study. Of the four undetected fusions, three eluded detection by all benchmarked tools. The remaining case involved a complex tripartite fusion, for which Fuscan successfully captured only one of the constituent breakpoints. The reduced sensitivity of Genefuse, relative to its reported performance, occurred primarily because we did not supplement its database with coordinates for common leukemia fusions, in contrast to the modifications made by Chen *et al.*

FACTERA exhibited highest specificity, producing false positives in only 3 samples, followed by Genefuse (18 samples). However, the lack of negative control data precluded the construction of a PoN for Fuscan. Consequently, Fuscan, alongside GRIDSS2, Manta, and DELLY, called false positives across all samples. Crucially, 92.75% (1037/1118) of Fuscan’s false-positive breakpoints were recurrent, underscoring the algorithm’s reliance on a PoN to efficiently filter systematic background noise.

### 3.4 Benchmarking Fuscan on running time

To evaluate computational efficiency, we measured execution times for all tools using 8 processes across 12 samples (1.2–14.0 GB), with each test performed in quintuplicate (Fig. 2C and Table 6, available as supplementary data at *Bioinformatics Advances* online). Total execution times included BWA-mem alignment and Picard deduplication for DELLY and FACTERA, and BWA-mem alignment for GRIDSS2 and Manta. Genefuse achieved the shortest turnaround time (2.42–13.65 min) by restricting its alignment to a specified gene list. GRIDSS2 (9.13–78.63 min) and Fuscan (6.89–91.87 min) demonstrated the highest efficiency, followed by FACTERA (8.99–101.16 min) and DELLY (11.29–106.41 min). In contrast, Manta required the longest processing time, ranging from 15.35 to 623.81 min. We further assessed scalability using 8 to 80 processes on a 10 GB dataset (Fig. 2C and Table 6, available as supplementary data at *Bioinformatics Advances* online), finding that Manta achieved the most significant speed improvement, while Fuscan and GRIDSS2 showed comparable scaling. In contrast, Genefuse’s analysis time remained largely unaffected by the number of processes, and speed gains for DELLY and FACTERA were limited. Overall, 32 processes represent the most cost-effective configuration for these tools, as further parallelization yielded diminishing performance returns.

## 4 Discussion

In oncology, targeted capture panel sequencing has become a standard procedure, with clinically validated tests (e.g. FoundationOne and MSK-IMPACT) extensively utilized across medical centers and certified diagnostic facilities (Zehir *et al.* 2017, Wang *et al.* 2025). Relative to WGS, these panel-based methods provide reduced expenses, enhanced sequencing depth, and superior analytical specificity (Milbury *et al.* 2022), permitting expansive utility in contexts such as liquid biopsy and cfDNA analysis (Clark *et al.* 2018). High-depth sequencing data inevitably introduce sequencing artifacts and mapping errors (Wong *et al.* 2014). Our benchmarking on clinical samples demonstrated that GRIDSS2, Manta, and DELLY, which were primarily developed for WGS data, generated a high number of false-positive calls. Although Manta allows for the input of matched germline samples to mitigate such noise, this requirement is often impractical in clinical settings where matched-normal DNA is frequently unavailable. In contrast, Fuscan implements a filtering strategy using a PoN constructed from 20 leukocyte samples. This approach excludes interference from artifacts and significantly reduces false positives caused by local alignments. However, the PoN strategy provided no performance improvement for GRIDSS2, FACTERA, Genefuse, or Manta, and only removed a limited number of false positives for DELLY.

Fuscan is specifically optimized for targeted sequencing data by restricting alignment to probe-covered regions designed for fusion capture, which maximizes the retrieval of true fusion events. Because the algorithmic design of Fuscan accounts for the distribution characteristics of hybridized DNA, it is incompatible with NGS data derived from multiplex PCR amplification. Fuscan is currently limited to detecting large structural variations (>1000 bp) and bipartite fusions between two distinct genes, rendering it unable to resolve complex rearrangements such as tripartite fusions. Regarding partner gene detection, Fuscan does not impose restrictions, similar to the approach used by FACTERA. This stands in contrast to Genefuse, which limits detection to predefined gene pairs. Although this restriction results in superior execution speed, it leads to the omission of fusions due to the diversity of fusion breakpoints. While DELLY was designed for WGS, its benchmarking performance trailed only Fuscan. It identified all true positives using its internal quality control, but this came at the cost of excessive false positives. This indicates that because WGS data have lower sequencing depth, these tools may require significantly higher thresholds when applied to high-depth targeted data.

We performed ROC analysis to compare the robustness of each tool. Fuscan proved to be the most robust with an AUC of 0.992, followed by DELLY at 0.974. If DELLY were to be applied to oncological drug diagnostics, it would likely require further optimization of the Youden index and more complex thresholding. Since the clinical significance of fusions varies by genomic region (Li *et al.* 2021), Fuscan allows for region-specific thresholds during the raw analysis stage. This feature makes Fuscan more suitable for oncological drug-response analysis as it can be directly implemented without additional parameter optimization.

## Supplementary Material

vbag152_Supplementary_Data

## Data Availability

The sequencing data for the reference standards have been deposited in CNGBdb (CNP0008423). The sequencing data for the clinical samples are subject to controlled access due to ethical restrictions. Please contact the corresponding author to request access to these data. The external leukemia targeted sequencing dataset analyzed in this study is publicly available from the original publication by Chen *et al.* (PRJNA1263052).

## References

[vbag152-B1] Bruno R , Fontanini G. Next generation sequencing for gene fusion analysis in lung cancer: a literature review. Diagnostics 2020;10:521. 32726941 10.3390/diagnostics10080521 PMC7460167

[vbag152-B2] Cameron DL , Baber J, Shale C et al GRIDSS2: comprehensive characterisation of somatic structural variation using single breakend variants and structural variant phasing. Genome Biol 2021;22:202. 34253237 10.1186/s13059-021-02423-x PMC8274009

[vbag152-B3] Chen B , Gao Z, Chen L et al Detection of leukemia gene fusions on DNA-level through targeted next-generation sequencing. PLoS One 2025;20:e0332407. 41066431 10.1371/journal.pone.0332407 PMC12510534

[vbag152-B4] Chen X , Schulz-Trieglaff O, Shaw R et al Manta: rapid detection of structural variants and indels for germline and cancer sequencing applications. Bioinformatics 2016;32:1220–2. 26647377 10.1093/bioinformatics/btv710

[vbag152-B5] Cheng DT , Mitchell TN, Zehir A et al Memorial Sloan Kettering-Integrated Mutation Profiling of Actionable Cancer Targets (MSK-IMPACT): a hybridization capture-based next-generation sequencing clinical assay for solid tumor molecular oncology. J Mol Diagn 2015;17:251–64. 25801821 10.1016/j.jmoldx.2014.12.006 PMC5808190

[vbag152-B6] Cingolani P , Platts A, Wang LL et al A program for annotating and predicting the effects of single nucleotide polymorphisms, SnpEff: SNPs in the genome of Drosophila melanogaster strain w1118; iso-2; iso-3. Fly (Austin) 2012;6:80–92. 22728672 10.4161/fly.19695 PMC3679285

[vbag152-B7] Clark TA , Chung JH, Kennedy M et al Analytical validation of a hybrid capture-based next-generation sequencing clinical assay for genomic profiling of cell-free circulating tumor DNA. J Mol Diagn 2018;20:686–702. 29936259 10.1016/j.jmoldx.2018.05.004 PMC6593250

[vbag152-B8] Cui M , Han Y, Li P et al Molecular and clinicopathological characteristics of ROS1-rearranged non-small-cell lung cancers identified by next-generation sequencing. Mol Oncol 2020;14:2787–95. 32871626 10.1002/1878-0261.12789 PMC7607175

[vbag152-B9] Jiang J , Wu X, Tong X et al GCC2-ALK as a targetable fusion in lung adenocarcinoma and its enduring clinical responses to ALK inhibitors. Lung Cancer 2018;115:5–11. 29290262 10.1016/j.lungcan.2017.10.011

[vbag152-B10] Kent WJ. BLAT - The BLAST-like alignment tool. Genome Res 2002;12:656–64. 11932250 10.1101/gr.229202 PMC187518

[vbag152-B11] Kohno T , Ichikawa H, Totoki Y et al KIF5B-RET fusions in lung adenocarcinoma. Nat Med 2012;18:375–7. 22327624 10.1038/nm.2644 PMC6430196

[vbag152-B12] Li H , Durbin R. Fast and accurate short read alignment with Burrows-Wheeler transform. Bioinformatics 2009;25:1754–60. 19451168 10.1093/bioinformatics/btp324 PMC2705234

[vbag152-B13] Li W , Guo L, Liu Y et al Potential unreliability of uncommon ALK, ROS1, and RET genomic breakpoints in predicting the efficacy of targeted therapy in NSCLC. J Thorac Oncol 2021;16:404–18. 33248323 10.1016/j.jtho.2020.10.156

[vbag152-B14] Lindeman NI , Cagle PT, Aisner DL et al Updated molecular testing guideline for the selection of lung cancer patients for treatment with targeted tyrosine kinase inhibitors guideline from the College of American Pathologists, the International Association for the Study of Lung Cancer, and the Association for Molecular Pathology. Arch Pathol Lab Med 2018;142:321–46. 29355391 10.5858/arpa.2017-0388-CP

[vbag152-B15] Merker JD , Oxnard GR, Compton C et al Circulating tumor DNA analysis in patients with cancer American Society of Clinical Oncology and College of American Pathologists Joint Review. Arch Pathol Lab Med 2018;142:1242–53. 29504834 10.5858/arpa.2018-0901-SA

[vbag152-B16] Milbury CA , Creeden J, Yip W-K et al Clinical and analytical validation of FoundationOne^®^CDx, a comprehensive genomic profiling assay for solid tumors. PLoS One 2022;17:e0264138. 35294956 10.1371/journal.pone.0264138 PMC8926248

[vbag152-B17] Newman AM , Bratman SV, Stehr H et al FACTERA: a practical method for the discovery of genomic rearrangements at breakpoint resolution. Bioinformatics 2014;30:3390–3. 25143292 10.1093/bioinformatics/btu549 PMC4296148

[vbag152-B18] Pan XK et al An analytical pipeline with multiple software tools improves detection of cancer-associated gene fusions from genomic DNA. J Mol Diagn 2024;26:140–9. 38008285 10.1016/j.jmoldx.2023.11.004

[vbag152-B19] Qiu X , You L, Wang C et al Non small cell lung cancer with SMARCA4 deficiency harboring rare EGFR mutations exhibited significant tumor response when treated with afatinib: a case report. Front Med 2025;19:170–3. 39808383 10.1007/s11684-024-1118-y

[vbag152-B20] Rausch T , Zichner T, Schlattl A et al DELLY: structural variant discovery by integrated paired-end and split-read analysis. Bioinformatics 2012;28:I333–I339. 22962449 10.1093/bioinformatics/bts378 PMC3436805

[vbag152-B21] Rodak O , Peris-Díaz MD, Olbromski M et al Current landscape of non-small cell lung cancer: epidemiology, histological classification, targeted therapies, and immunotherapy. Cancers (Basel) 2021;13:4705. 34572931 10.3390/cancers13184705 PMC8470525

[vbag152-B22] Rosenbaum JN , Bloom R, Forys JT et al Genomic heterogeneity of ALK fusion breakpoints in non-small-cell lung cancer. Mod Pathol 2018;31:791–808. 29327716 10.1038/modpathol.2017.181

[vbag152-B23] Soda M , Choi YL, Enomoto M et al Identification of the transforming EML4-ALK fusion gene in non-small-cell lung cancer. Nature 2007;448:561–6. 17625570 10.1038/nature05945

[vbag152-B24] Solomon BJ , Mok T, Kim D-W, et al; PROFILE 1014 Investigators. First-Line crizotinib versus chemotherapy in ALK-positive lung cancer. N Engl J Med 2014;371:2167–77. 25470694 10.1056/NEJMoa1408440

[vbag152-B25] Takeuchi K , Soda M, Togashi Y et al RET, ROS1 and ALK fusions in lung cancer. Nat Med 2012;18:378–81. 22327623 10.1038/nm.2658

[vbag152-B26] Wang Y , Wang X, Huang X-Y et al A prognostic index integrating deep learning baseline PET/CT biomarkers and multi-omics profiling in diffuse large B cell lymphoma. Cell Rep Med 2025;6:102452. 41260205 10.1016/j.xcrm.2025.102452 PMC12711681

[vbag152-B27] Wong SQ , Li J, Tan AY-C et al; CANCER 2015 Cohort. Sequence artefacts in a prospective series of formalin-fixed tumours tested for mutations in hotspot regions by massively parallel sequencing. BMC Med Genomics 2014;7:23. 24885028 10.1186/1755-8794-7-23 PMC4032349

[vbag152-B28] Wu G , Guo L, Gu Y et al The genomic characteristics of RET fusion positive tumors in Chinese non-small cell lung cancer (NSCLC) patients. J Cancer Res Clin Oncol 2023;149:1019–28. 35220468 10.1007/s00432-022-03959-6 PMC9984339

[vbag152-B29] Zehir A , Benayed R, Shah RH et al Mutational landscape of metastatic cancer revealed from prospective clinical sequencing of 10,000 patients. Nat Med 2017;23:703–13. 28481359 10.1038/nm.4333 PMC5461196

